# ﻿*Centaurealovricii*, a new species of ﻿*C.* sect. ﻿*Centaurea* (Asteraceae) from Croatia

**DOI:** 10.3897/phytokeys.214.89404

**Published:** 2022-11-30

**Authors:** Sandro Bogdanović, Igor Boršić, Ivica Ljubičić, Salvatore Brullo, Gianpietro Giusso del Galdo

**Affiliations:** 1 University of Zagreb, Faculty of Agriculture, Department of Agricultural Botany, Svetošimunska 25, 10000 Zagreb, Croatia University of Zagreb Zagreb Croatia; 2 Centre of Excellence for Biodiversity and Molecular Plant Breeding, Svetošimunska 25, 10000 Zagreb, Croatia Centre of Excellence for Biodiversity and Molecular Plant Breeding Zagreb Croatia; 3 Institute for Environment and Nature, Ministry of Economy and Sustainable Development, Radnička cesta 80/7, 10000 Zagreb, Croatia Institute for Environment and Nature, Ministry of Economy and Sustainable Development Zagreb Croatia; 4 Department of Biological, Geological and Environmental Sciences, University of Catania, Via A. Longo 19, 95125 Catania, Italy University of Catania Catania Italy

**Keywords:** Adriatic region, Balkan flora, *Centaurea*, Croatia, morphology, new species, taxonomy

## Abstract

A new species, *Centaurealovricii*, is described and illustrated from the island of Vis (Dalmatia, Croatia). It occurs on northwest-facing calcareous cliffs near the sea, where it grows with several other rare endemic species. *Centaurealovricii* is morphologically similar to *C.glaberrima* and *C.divergens* of C.sect.Centaurea, from which it differs in having more succulent leaves with larger and less incised leaflets, bigger capitula, larger phyllaries with more developed appendages and denser and undulate fimbriae, larger florets, bigger achenes, and longer pappus. Its morphological features, distribution, ecology, conservation status and taxonomic affinities are examined. In addition, a new iconography and lectotypification for *C.glaberrima* and *C.divergens* is provided.

## ﻿Introduction

The genus *Centaurea* L. is one of the largest genera in the family Asteraceae. In its current circumscription as a natural group, it includes about 250 species (Susanna and Garcia-Jacas 2007, 2009). It is mainly distributed in the Euro-Mediterranean and south-western Asian territories (Susanna et al. 1995, 2006; Susanna and Garcia-Jacas 2007, 2009; Font et al. 2009; López et al. 2011; Hilpold et al. 2014a, b). As emphasised by Hilpold et al. (2014b), three subgenera can be recognised, namely C.subgen.Centaurea, C.subgen.Lopholoma (Cass.) Dobrocz., and C.subgen.Cyanus (Mill.) Cass. ex Hayek, each represented by numerous sections and subsections.

According to the literature (Hilpold 2012; Boršić 2013; Hilpold et al. 2014a, b), within sect. Centaurea of subgen. Centaurea three subsections are recognised, i.e. subsect. Centaurea, subsect. Phalolepis (Cass.) Garcia-Jacas, Hilpold, Susanna & Vilatersana, and subsect. Willkommia (Blanca) Garcia-Jacas, Hilpold, Susanna & Vilatersana. In particular, subsect. Centaurea [formerly sect. Acrolophus (Cass.) DC.] is characterised by having triangular phyllary appendages that are regularly ciliate at the margin and often provided with a mucro at the apex. This subsection is widespread in the Mediterranean area (Hilpold 2012). Several of its species are distributed in Croatia, Montenegro, Serbia, Bosnia and Herzegovina, while some of them are exclusively known from Croatia and its islands (Lovrić 1976, 1990, 1995; Nikolić et al. 2015; Nikolić 2020, 2022). Within this subsection several endemic species are known from the western Balkan peninsula: *C.biokovensis* Teyber s.l., *C.crithmifolia* Vis., *C.cuspidata* Vis. s.l., *C.dalmatica* A.Kern., *C.derventana* Vis. & Pančić, *C.divergens* Vis., *C.fridericii* Vis., *C.glaberrima* Tausch, *C.gloriosa* Radić, *C.incompta* Vis., *C.kartschiana* Scop., *C.radichii* Plazibat, *C.spinosociliata* Seenus s.l. and *C.visianiana* Plazibat.

During field investigations focused on the flora of the Dalmatian islands (Croatia) a peculiar chasmophilous population of *Centaurea* growing on the cliffs of the island of Vis was found. Previously, it was examined by Lovrić (1982, 1983, 1990, 1995) and named *C.issaea* or C.glaberrimavar.issaea. However, these names must be considered *nomina nuda*, because a description was not provided for them and no type material was indicated. According to Art. 38.1 and 40 of the ICN (Turland et al. 2018), the names were therefore not validly published. Later, these names were used by several authors (Van der Maarel and Van der Maarel-Versluys 1996; Bogdanović and Ruščić 2011; Boršić 2013; Terzi et al. 2019; Nikolić 2020, 2022), all confirming the occurrence of this taxon on the island of Vis. According to the results of molecular genetic investigations (nuclear and plastid DNA sequences, as well as Amplified Fragment Length Polymorphism data - AFLP) carried out by Boršić (2013) on the amphi-Adriatic species of *Centaurea*, the population from Vis island is closely related to *C.glaberrima*, a species occurring in southern Croatia, Montenegro, Albania, Bosnia and Herzegovina. To examine the affinities of these two taxa, morphological studies of populations from the island of Vis and the type locality of *C.glaberrima* in Dalmatia were carried out, as well as of its type specimen kept in PRC. These analyses show that the population from the island of Vis is highly distinct from *C.glaberrima*, as well as from the allied *C.divergens* and the other species belonging to subsect. Centaurea. Therefore, based on morphological and ecological differences with other species in this subsection, the plants in question are treated here as a new species for science and named *C.lovricii*.

## ﻿Materials and methods

For this morphological study, we examined 90 herbarium specimens and scanned images of specimens from several, also virtual, herbaria (BEO, BEOU, BP, BUNS, CAT, CNHM, K, MW, P, PAD, PI, PRC, SARA, U, W, WU, ZA, ZAGR and ZAHO). The herbarium acronyms follow Thiers (2022). In addition, we collected 20 plants of *C.lovricii* from the island of Vis (one population), and 50 plants of *C.glaberrima* (seven populations) and 40 plants of *C.divergens* (seven populations) from several other localities in Croatia, including *loci classici*. The investigations were carried out on material preserved in alcohol and glycerine solution, as well as on herbarium exsiccatae deposited at CAT and ZAGR. Information about distribution, habitat, ecology and phenology were obtained from field data and herbarium specimens.

## ﻿Taxonomic treatment

### ﻿1. Centaurea lovricii

Taxon classificationPlantaeAsteralesAsteraceae

Bogdanović, Boršić, Ljubičić, Brullo & Giusso sp. nov.

E9F3550B-341D-59EF-B641-72733B71BB37

urn:lsid:ipni.org:names:77309067-1

**Figure 1.**
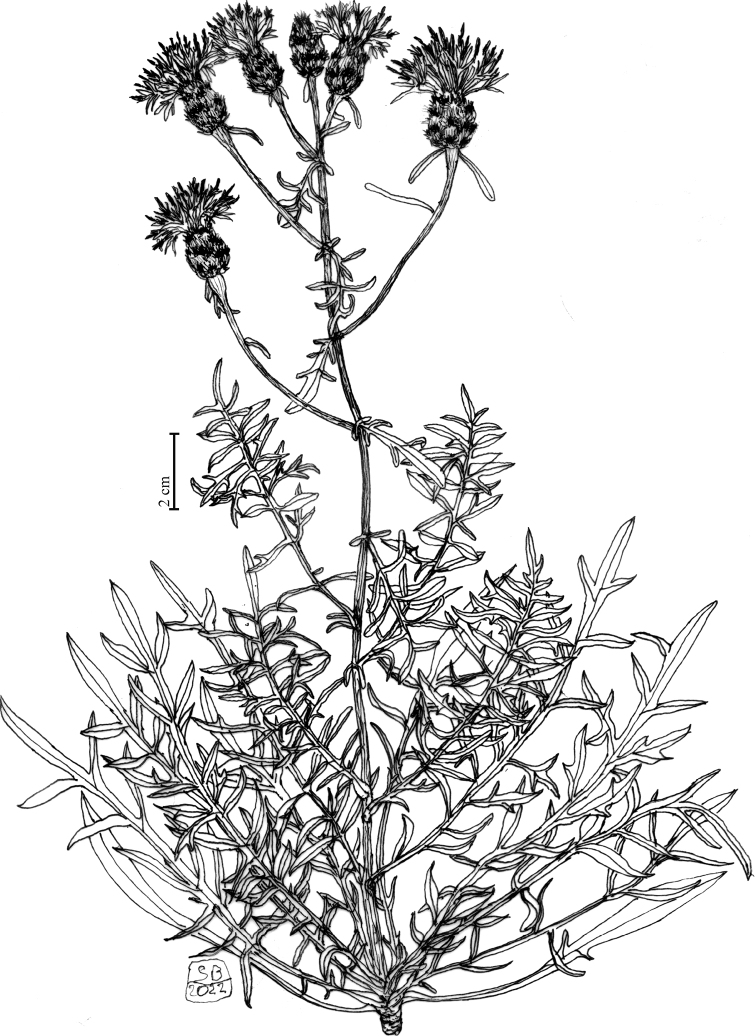
Habit of *Centaurealovricii* sp. nov. Drawn by Salvatore Brullo.

**Figure 2.**
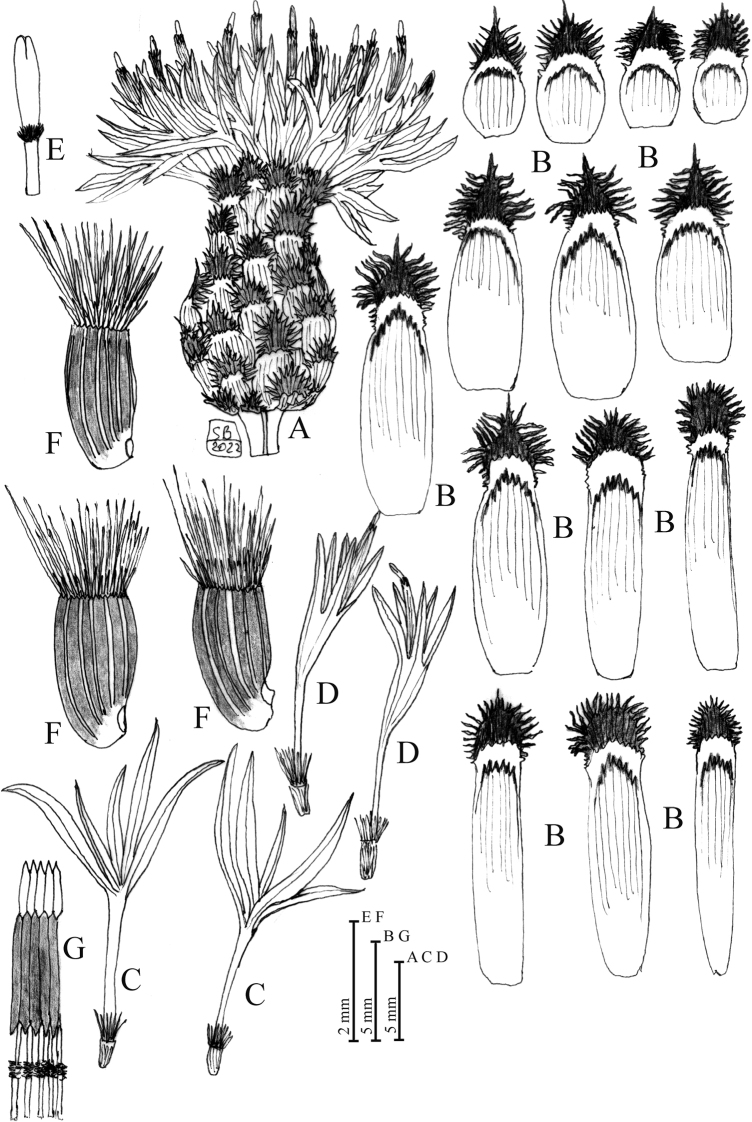
*Centaurealovricii* sp. nov. **A** capitulum **B** phyllaries **C** outermost sterile florets **D** disc florets **E** stigma **F** achenes **G** stamens upper part. Drawn by Salvatore Brullo.

**Figure 3.**
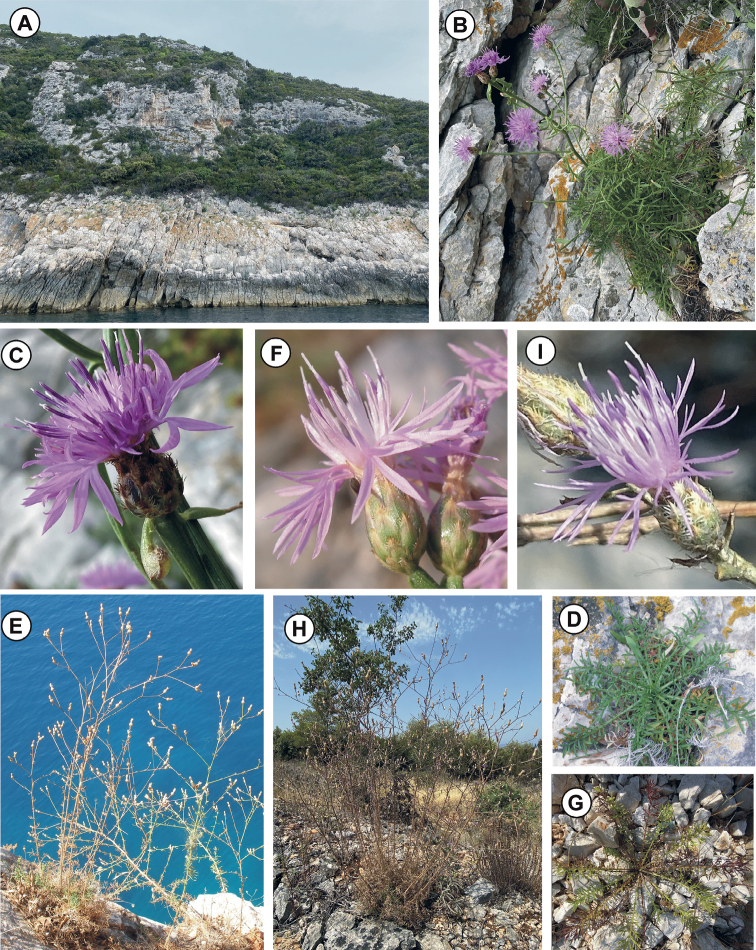
*Centaurealovricii* sp. nov. **A** habitat **B** plant growing in natural habitat **C** capitulum **D** basal leaves; *C.glaberrima***E** plant growing in natural habitat **F** capitula **G** basal leaves; *C.divergens***H** plant growing in natural habitat **I** capitulum. Photographed by Sandro Bogdanović.

Figs 1 , 2 , 3B, C, D

Centaurea glaberrima var. issaea Lovrić, Taxon 31(4): 763, 1982 (nom. nud.).
Centaurea issaea Lovrić, Pos. izd. Muz. grada Šibenika 10: 191, 1983 (nom. nud.).

#### Type.

Croatia. Island of Vis, Blišćevac bay, calcareous rocky slopes, 16 May 2021, 43°4'23.30"N, 16°5'51.59"E, *S. Bogdanović & M. Temunović s.n.* (holotype: ZAGR–68512; isotypes: CAT, CNHM, ZA, ZAGR).

#### Description.

Perennial herb, with several stems, sterile leaf-rosettes and woody rootstock. Stems erect, glabrous, striate, shiny green, 25–50 cm long, not winged, laxly branched. Basal leaves fleshy, shiny green, glabrous, 1–2 pinnatisect, 7–20 cm × 5–40 mm; leaflets 5–15 per side, lanceolate, linear-lanceolate to linear, or oblong lanceolate, 5–30 × 0.8–5(–10) mm, often the terminal ones lanceolate and up to 10 mm wide; young leaves oblong lanceolate with entire margin or with few lateral teeth; petioles 5–55 mm long. Median and upper cauline leaves similar to basal ones, sessile or subsessile, 1(-2) pinnatisect, 1.5–12 × 0.5–3 cm; leaflets 1–12 per side, linear-lanceolate to linear, margins entire. Synflorescence laxly paniculate, with 7–30 solitary capitula. Peduncles 1–20 cm long, apex clavate, bracts 1–8, entire. Involucre ovoid, 13–15 × 8–12 mm. Phyllaries straw-coloured, glabrous, coriaceous, dorsally with 3–7 nerves; the outer ones ovate, 6.5–8 × 2.5–3.5 mm; the median ones ovate-oblong, 10–15 × 3.5–4.5 mm; the innermost linear, 12.5–15 × 2–2.8 mm; phyllary appendages well-developed, triangular to orbicular, dark brown, appressed, densely fimbriate with 7–10 pairs of undulate fimbriae, 0.3–1.7 mm long, ending with a terminal acute tooth. Florets pink-purplish. Outermost florets sterile; corolla tube glabrous, 7.5–9 mm long; corolla lobes linear, irregular, 7.5–11.5 mm long. Disc florets fertile; corolla tube 10–11.5 mm long; corolla lobes linear, equal, 5 mm long. Stamens 12.5–13.5 mm long; filaments 4.5–5 mm long; anthers 8–8.5 mm long, dark violet. Style 16–16.5 mm long, ciliate at apex; stigmas 1.5 mm long. Achenes oblong, 2.6–3 × 1.4–1.5 mm, glabrous, dark brown up to the apex, with several irregular straw-coloured longitudinal ridges. Pappus obscurely biseriate, whitish, with bristles 0.6–2.3 mm long.

#### Etymology.

This species is dedicated to the Croatian botanist Andrija–Željko Lovrić (1943–2018), who was the first to collect it and consider it as a new species.

#### Phenology.

*Centaurealovricii* flowers from May to late June, and fruits from late June to July.

#### Distribution and ecology.

**Figure 4.**
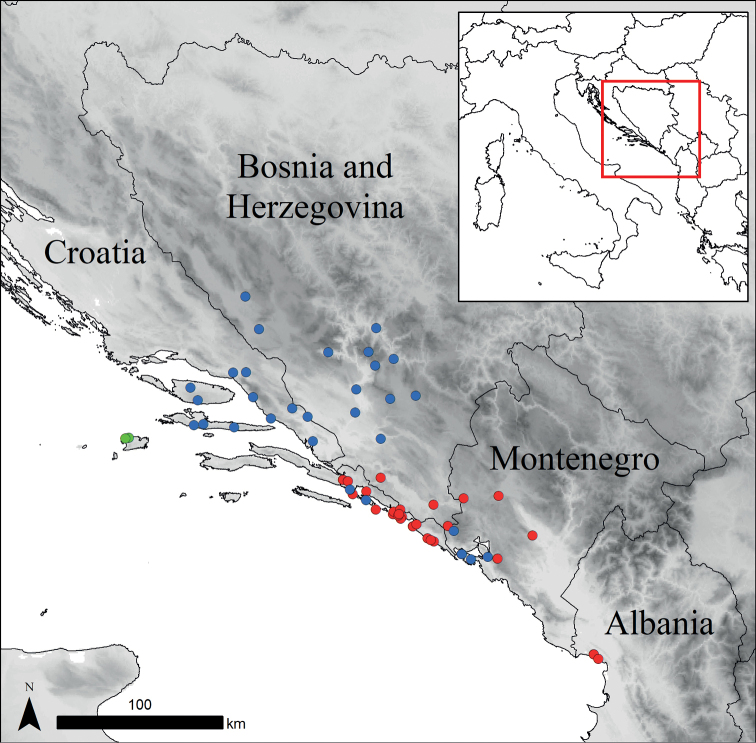
Distribution of *Centaurealovricii* sp. nov. (green dots), *C.glaberrima* (red dots) and *C.divergens* (blue dots).

This new species grows along the northern coast between Dragodid Bay and Oključina Bay of the island of Vis in Dalmatia, Croatia (Fig. 4). It grows on sea facing cliffs constituted of Triassic dolomites (Lozić et al. 2012) at 10–100 m a.s.l., in rocky crevices together with many other rare or endemic species (Fig. 3A). The most frequent chasmophytes occurring in this habitat are Aurinialeucadeasubsp.scopulorum (Ginzb.) Plazibat, *Campanulateutana* Bogdanović & Brullo, *Centaurearagusina* L., Helichrysumitalicumsubsp.pseudolitoreum (Fiori) Bacch., Brullo & Mossa, *Brassicaincana* Ten., *Limoniumissaeum* Bogdanović & Brullo, Pimpinellatragiumsubsp.lithophila (Schischk.) Tutin and *Sesleriainterrupta* Vis. (Bacchetta et al. 2003; Bogdanović and Ruščić 2011; Bogdanović et al. 2014; Bogdanović and Brullo 2015; Terzi et al. 2019).

#### Conservation status.

The single population of *C.lovricii* is composed of fewer than 1000 scattered mature individuals, so it can be considered Vulnerable (VU D1) according to the IUCN Red List Categories and Criteria (IUCN 2022). It is currently distributed in a very narrow coastal belt of ca. 0.5 km^2^, so it has a restricted area of occupancy and only one location, but as its growing site is very steep and quite inaccessible, which makes the population unthreatened by any human disturbance, it does not qualify for subcriterion D2.

#### Chromosome number.

According to Lovrić (1982, 1983) and Van Loon (1987) the ploidy level of *C.lovricii*, previously attributed to “C.glaberrimavar.issaea”, is tetraploid with 2*n* = 4*x* = 36 chromosomes, similarly to that of the allied species *C.glaberrima* and *C.divergens* (Siljak-Yakovlev et al. 2005). According to Boršić (2013) the estimated ploidy level for “*C.issaea*” is also tetraploid.

#### Discussion.

**Figure 5.**
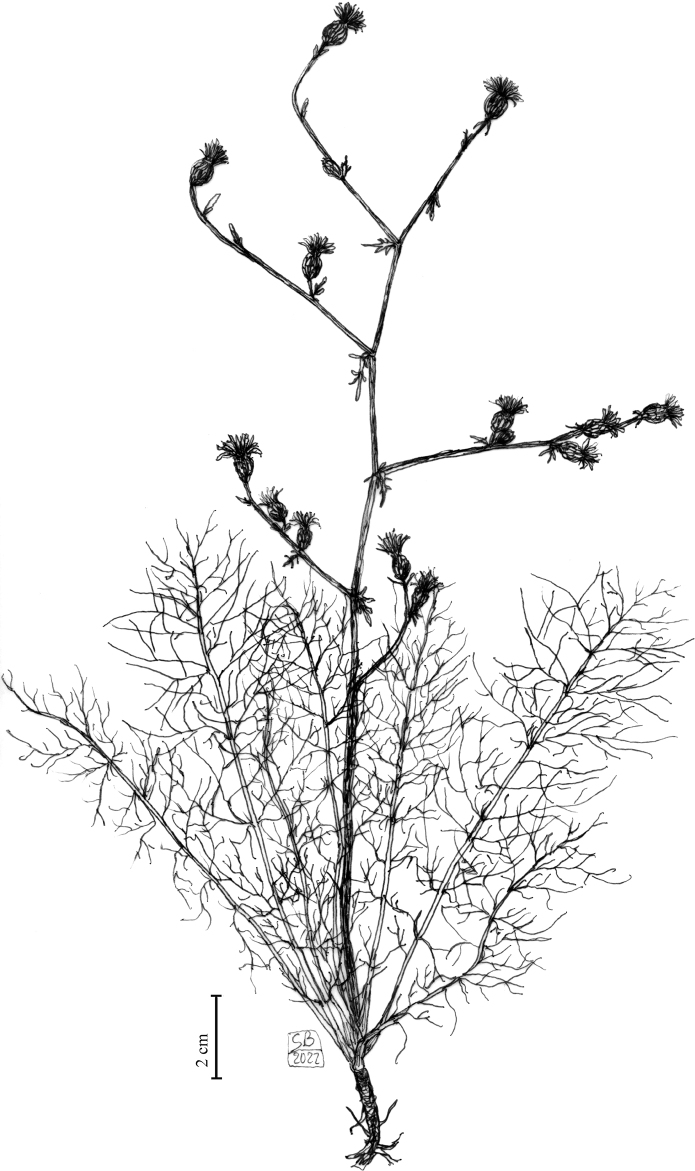
Habit of *Centaureaglaberrima*. Drawn by Salvatore Brullo.

**Figure 6.**
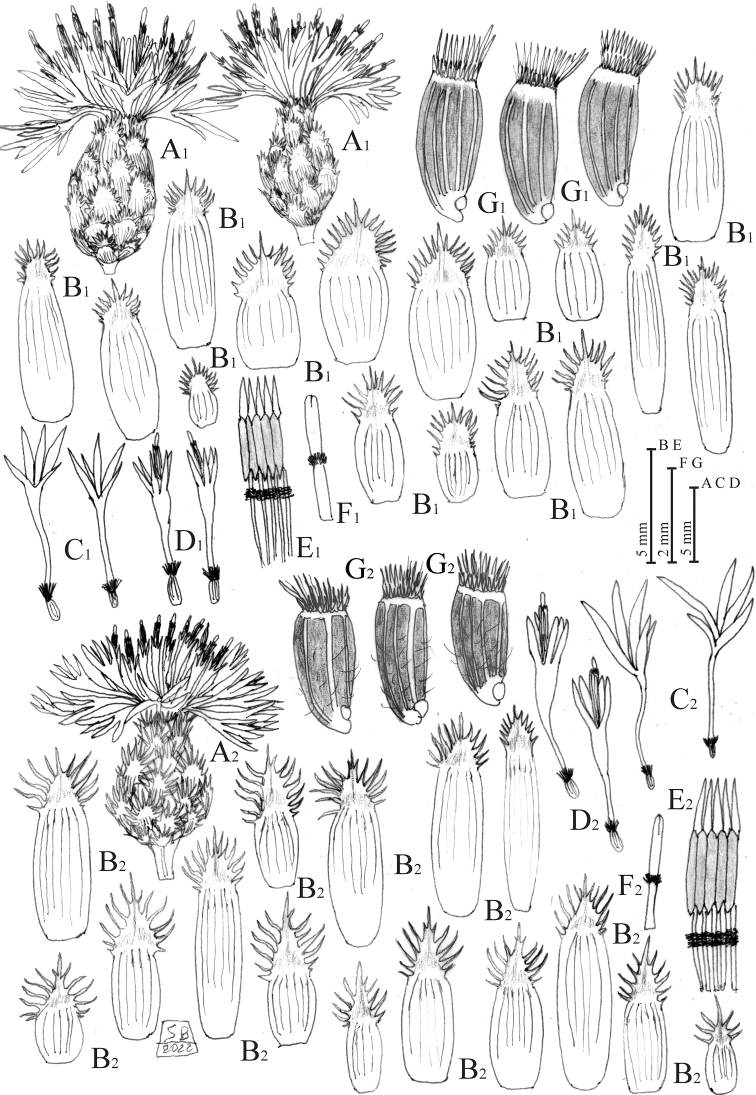
*Centaureaglaberrima* (1) and *C.divergens* (2) **A** capitula **B** phyllaries **C** outermost sterile florets **D** disc florets **E** stamens upper part **F** style.

Within Centaureasubsect.Centaurea, *C.lovricii* is most similar to *C.glaberrima*, particularly in the morphology of its capitula and phyllaries. *Centaureaglaberrima* is a species occurring in several localities in the north-western Balkans (Nikolić et al. 2015). However, these two species show some significant differences in habit and the morphology of their leaves, capitula and achenes (Table 1). In particular, *C.glaberrima* (Figs 5, 6) has a more robust and rigid habit, stems with up to 80 capitula, rigid basal leaves with leaflets linear-filiform (0.5–2 mm wide), involucre 6–10 mm long, with phyllaries up to 8 mm long, appendages straw-coloured, often tinged with pale-brown, with sparse fimbriae, outermost sterile florets with corolla tube 6–6.5 mm long and pappus bristles 0.2–0.8 mm long. In addition, the two species have different ecological requirements. *Centaureaglaberrima* usually behaves as a ruderal plant growing along roadsides, in dry rocky grasslands and occasionally also in rupestrian stands, whereas *C.lovricii* is a true chasmophyte in coastal stands. A population genetic study of some endemic Adriatic species of *Centaurea* using AFLPs was carried out by Boršić (2013), who showed the distinctness of the population from the island of Vis (sub *C.issaea*) from *C.glaberrima*, but also concluded that this population shows evidence of introgression with *C.glaberrima* and *C.spinosociliata*. A phylogeny based on nrDNA internal transcribed spacer sequences revealed that *C.lovricii* still has an unclear phylogenetic position among the species of C.sect.Centaurea included in this study (Boršić 2013).

**Table 1.** Main diagnostic features of *Centaurealovricii*, *C.glaberrima* and *C.divergens*.

	*C.lovricii*	*C.glaberrima*	*C.divergens*
Stem	glabrous, shiny, 25–50 cm long,	glabrous, dull, 25–80 cm long,	hispid, dull, 25–100 cm long,
Basal leaves	fleshy, robust, glabrous, 1–2 pinnatisect, 7–20 cm long, with leaflets lanceolate to linear, 5–30 × 0.8–5 mm, the terminal one up to 10 mm wide	thin, rigid, glabrous, 1–3 pinnatisect, 8–15 cm long, with leaflets linear-filiform, 2–15 × 0.5–0.8(–2) mm	thin, rigid, hispid, 1–3 pinnatisect, 8–20 cm long, with leaflets linear-filiform, 2–15 × 0.5–0.8(–2) mm
Cauline leaves	1.5–12 cm long, 1–12 leaflets per side, linear lanceolate to linear	0.5–5 cm long, 1–15 leaflets per side, linear-filiform	0.5–7 cm long, 1–10 leaflets per side, linear-filiform
Synflorescence	laxly branched, with 7–30 capitula	laxly paniculate, very branched, with (15)50–80 capitula	densely paniculate, very branched, with 15–80 capitula
Capitula	solitary, involucre 13–15 × 8–12 mm, with peduncle 1–20 cm long	solitary to grouped, involucre 6–8.5(–10) × 4.5–5.5 mm, with peduncle 0.3–6 cm long	solitary to grouped, involucre 6–10 × 4–6.5 mm, with peduncle 0.2–7 cm long
Phyllaries	6.5–15 × 2–4.5 mm, with appendages triangular to orbicular, dark-brown, densely fimbriate with 7–10 pairs of undulate fimbriae 0.3–1.7 mm long	2.5–8 × 1.2–2.6 mm, with appendages triangular to orbicular, straw-coloured often tinged with pale-brown, 4–8 pairs of lax and more or less straight fimbriae, which are 0.3–1(–1.5) mm long	3–8 × 1.4–2.2 mm, with appendages triangular, straw-coloured, tinged with pale-brown, 3–6 pairs of lax and more or less straight fimbriae, which are 0.2–2 mm long
Corolla of outermost sterile florets	tube 7.5–9 mm long, lobes 7.5–11.5 mm long	tube 6–6.5 mm long, lobes 2–4.1 mm long	tube 5.5–6 mm long, lobes 3–6 mm long
Corolla of disc florets	tube 10–11.5 mm long, lobes 5 mm long	tube 5–6 mm long, lobes 2–2.5 mm long	tube 6.5–7.5 mm long, lobes 3–3.3 mm long
Stamens	12.5–13.5 mm long, with filament 4.5–5 mm long and anthers 8–8.5 mm long	7.5–9.8 mm long, with filaments 5.3–5.8 mm long and anthers 2.2–4 mm long	10–10.5 mm long, with filaments 5–5.5 mm long and anthers 5 mm long
Style	16–16.5 mm long	7.5–8.3 mm long	10 mm long
Stigma	1.5 mm long	1.25 mm long	1.2 mm long
Achenes	2.6–3 × 1.4–1.5 mm, glabrous, dark-brown up to the apex	2.5–2.7 × 1–1.2 mm, glabrous, dark-brown with straw-coloured strip above	2.3–2.5 × 0.9–1.1 mm, sparsely hairy, dark-brown with straw-coloured strip above
Pappus	obscurely 2-seriate, with bristles 0.6–2.3 mm long	2-seriate, with bristles 0.2–0.8 mm long	2-seriate, with bristles 0.2–1.1 mm long

Another morphologically similar species is *C.divergens* (Fig. 6), which is often treated as a subspecies or variety of *C.glaberrima* (Malý 1928; Hayek 1931; Lovrić 1967–1968; Dostál 1976; Gavrilović and Janaćković 2022), but its treatment as a distinct species, as proposed by Visiani (1847) and Hayek (1901), seems to be more appropriate (see Table 1). It differs from *C.lovricii* in having a hispid stem, rigid and dull green basal leaves with linear-filiform leaflets (0.5–2 mm wide), a 6–10 mm long involucre, phyllaries that are up to 8 mm long, with up to 2 mm long fimbriae, outermost sterile florets with a 5.5–6 mm long corolla tube, and up to 1.1 mm long pappus bristles. They are also ecologically well differentiated, because *C.divergens* is usually a typical ruderal species, while *C.lovricii* is a chasmophyte.

A nomenclatural overview and lectotype designation for *C.glaberrima* and *C.divergens*, species closely related to *C.lovricii*, are here provided:

### ﻿2. Centaurea glaberrima

Taxon classificationPlantaeAsteralesAsteraceae

Tausch, Syll. Pl. Nov. 2: 249, 1828.

D89FFCFC-467A-5A4C-8BAE-ECFDD3503D16

Figs 5 , 6

Centaurea punctata Vis., Flora 12: 23, 1829. Type: Croatia. In agris sterilibus Dalm. [Dalmatia] montanae (prope Duare), *Visiani s.n.* (lectotype, designated here: PAD – HD02644).

#### Type.

Croatia. e Dalmat. [Dalmatia], s.d., *Sieber s.n.* (lectotype, central specimen designated here: PRC).

#### Nomenclatural note.

Tausch (1828) described *C.glaberrima* from Dalmatia without indicating any precise locality. The herbarium sheet, with three specimens, that we found in PRC has an original herbarium label with Tausch’s handwriting: “*Centaurea* – ?, *e Dalmat. Sber*. *capit: parvus ovoideus divaricatum*”. Tausch usually shortened geographical names: “e Dalmat.” refers to specimens collected in eastern Dalmatia, while “Sber.” refers to the collector (i.e. Sieber). In addition, he provided a short morphological description. Later, to the same herbarium label, the identification [= *C.punctata* Vis.] was added. This was probably done by V. Kosteletzky (pers. observ. by curator P. Mraz). The fourth specimen on the sheet belongs to *C.spinosociliata* and it is correctly identified on a separate herbarium label by A. Hayek. Here we select the central specimen, which fits Tausch’s description, as lectotype for the name *C.glaberrima*. One year later, Visiani (1829) described *C.punctata* from Duare [Zadvarje] in Dalmatia. We consulted the type specimen in PAD (PAD – HD02644), which fits Visiani’s description given in the protologue. In fact, the morphology of the specimen perfectly corresponds to Tausch’s description of *C.glaberrima*. Therefore, we here designate this specimen as a lectotype and include the name *C.punctata* as a synonym of *C.glaberrima*.

#### Iconography.

Figs 5, 6; Tav. 11, Visiani, Fl. Dalmat. 2: 39, 1847, sub *C.punctata*; Tav. 47 (II, III, 4–14), Reichenbach, Icon. Fl. Germ. Helv. 15: 30, 1852, sub *C.punctata*; Tav. 155 (II, 10–11), Reichenbach, Icon. Fl. Germ. Helv. 15: 31, 1853, sub *C.punctata*.

#### Distribution.

According to Greuter (2006), Barina et al. (2015) and Nikolić et al. (2015), *C.glaberrima* is distributed in Albania, Bosnia and Herzegovina, Croatia and Montenegro (Fig. 4).

### ﻿3. Centaurea divergens

Taxon classificationPlantaeAsteralesAsteraceae

Vis., Fl. Dalmat. 2: 37, 1847.

A0E3B4D3-75EB-5259-9709-B6B3A98F3F4F

Fig. 6

Acosta divergens (Vis.) Soják, Čas. Nár. Muz. Odd. Přir. Prague 140: 134, 1972. Type. Based on Centaureadivergens Vis.
Centaurea glaberrima subsp. divergens (Vis.) Hayek, Repert. Spec. Nov. Regni Veg. Beih. 30(2): 260, 1931. Type. Based on Centaureadivergens Vis.
Centaurea glaberrima var. divergens (Vis.) Malý, Glasn. Zemaljsk. Muz. Bosne Hercegovine Sarajevu. Prir. Nauke 40(1): 122, 1928 Type. Based on Centaureadivergens Vis.
Centaurea petteri Rchb.f., Icon. Fl. Germ. Helv. 15: 36, 1852. Type. Croatia. Auf dem Monte Mossor Dalm., June, *Reichenbach fil. s.n.* (W1889-0292916).

#### Type.

Croatia. In apricis montium Lesina, s.d., *Stalio 448* (lectotype, designated here: PAD – HD02637).

#### Nomenclatural note.

*Centaureadivergens* was described from the island of Hvar in central Dalmatia by Visiani (1847). In the protologue, Visiani clearly indicated “*in saxosis collium, et montium circa Lessina, unde misit prof. Stalio*” and we found original type material in the PAD herbarium that corresponds to description given in the protologue. Here we select a specimen that fits Visiani’s description as lectotype for the name *C.divergens*.

#### Iconography.

Fig. 6; Tav. 12(b)(41), Visiani, Fl. Dalmat. 2: 37, 1847; Tav. 51 (772) I, 1–7, Reichenbach, Icon. Fl. Germ. Helv. 15: 35, 1852; Tav. 52 (783) II, 9–16, Reichenbach, Icon. Fl. Germ. Helv. 15: 36, 1852, sub *Centaureapetteri*.

#### Distribution.

According to Greuter (2006), *C.divergens* is distributed in Bosnia and Herzegovina, Croatia, Montenegro (Fig. 4) and as an adventive plant in France.

#### Additional specimens examined.

***Centaurealovricii* (paratypes): Croatia.** Split-Dalmatia County, island of Vis, Kraljičina špilja, vertical rocks, 26 April 2010, *S. Bogdanović & Z. Liber s.n.* (ZAGR); Dalmazia, Isola di Vis, Oključina, pereti rocciosi sopra il mare, 25 May 2011, *S. Bogdanović, S. Brullo & G. Giusso s.n.* (CAT, ZAGR); Island of Vis, Oključina, vertical cliffs, 12 June 2010, *S. Bogdanović & I. Boršić s.n.* (ZAGR); Island of Vis, Oključina, vertical cliffs, 23 May 2010, *S. Bogdanović s.n.* (ZAGR). ***Centaureaglaberrima*: Albania.** District of Shkodër, Rera e Hedhur E of village Baks-Rrjoll, on the slope of Mts Mali i Rencit, on limestone rocks, 41.83324°N, 19.53921°E, 76 m a.s.l., 5 August 2011, *Z. Barina & G. Somogyi 19743* (BP761381); District of Shkodër, in mountain Maja e Zezë above village Baks-Rrjoll, in dry grassland, on limestone, 41.85733°N, 19.50846°E, 248 m a.s.l.; 4 May 2014, *Z. Barina, D. Pifkó & G. Puskás 23227* (BP767834). **Croatia.** Flora Dalmatica, In rupibus promontorii Punta Spezerea pr. Ragusam, 5 June 1906, *A. Degen s.n.* (PI012666); E Dalmat, s.d., *Tausch s.n.* (PRC); Dalmatia, 1832, *R. Visiani s.n.* (K000772946); Južna Hrvatska, Velji Do, obronci planina iznad Cavtata, 22 July 2013, *S. Bogdanović & I. Boršić s.n.* (ZAGR44365); Ragusa in Dalmatia, *R. Visiani s.n.*, sub *C.punctata* Vis. (P02815194); Ragusa, 15 June 1867, *R. Huter s.n.*, sub *C.punctata* Vis. (P02815195, P02815196, P02815197); Raguse, 14 June 1861, *R. Huter s.n.* (P04215180); Pelješac, Ston, uz cestu, 30 July 2021, *S. Bogdanović s.n.* (CAT, ZAGR); Pelješac, Ston, Majkovi, 11 July 2021, *S. Bogdanović s.n.* (CAT, ZAGR); Južna Dalmacija, otok Olipa, južna strana, uz obalu mora, 10 May 2018, *S. Bogdanović & I. Rešetnik s.n.* (ZAGR50465, ZAGR50466, ZAGR50462, ZAGR50463); Dalmacija, otok Lopud, stijene uz obalu mora, 12 May 2018, *I. Rešetnik & S. Bogdanović s.n.* (ZAGR53125); Dubrovnik, kod hotela Belvedere, stijene uz obalu mora, 12 May 2018, *I. Rešetnik & S. Bogdanović s.n.* (ZAGR53144); Otok Šipan: Kaludrica, u garigu, 25 August 1979, *M. Hećimović s.n.* (ZA13511); Na morskoj litici, Vrtac kod sela Popovići, ca 50 m, 19 July 1926, *V. Loschnigg s.n.* (ZA13510); In colle Gorizae et ad margines agrarum olivat. Lapad, 1868, *M. Vodopić s.n*. (ZA13508), sub *C.punctata* Vis.; Otok Lokrum: travnjak u masliniku kraj zgrade, 2 May 1959, *S. Horvatić s.n.* (ZA1307), sub *C.punctata* Vis.; In saxosis fruticetis sempervirentis ad Lapad (Gruž), 28 July 1928, *Th. Soška s.n.* (MW0794564); Slano, February 1972, *M. Obradović s.n.* (BUNS18767), Gradac, 9 May 1967, *M. Obradović s.n.* (BUNS18956); In rupibus calc. Ombla, pr. Dubrovnik, 15 August 1946, *O. Grebenščikov s.n.* (BEO26342); Lokrum, *s.d.*, *S. Jovanović s.n.* (BEOU37269); Dalmatia. In rupestribus apricis prope urbem Dubrovnik (Ragusa), *s.d.*, *A. Latzel s.n.* (BEOU37274); Dalmatia merid., in saxosis calcareis montis Ilinovo Brdo supra Gruda, 13 July 1928, *Soška s.n.* (BEOU37276); Dalmatia merid., in saxosis calcareis montis Srdj supra Dubrovnik, 20 July 1928, *Soška s.n.* (BEOU37277); Srdj (Dubrovnik), krečnjački kamenjari, 25 May 1990, *S. Stevanović & S. Jovanović s.n.* (BEOU502.90); Dubrovnik (okolina), Srđ, 25 May 1988, *S. Jovanović s.n.* (BEOU719); Padine brda Srđ, Dubrovnik, kamenjarski travnjaci, October 1993, *S. Maslo s.n.* (ZAGR); Konavoske stijene, Dubrovnik, pukotine krečnjačkih stijena uz morsku obalu, 16 July 2003, *S. Maslo s.n.* (ZAGR); Konavoske stijene (najzapadniji dio, kod Popovića), kamenjare, 1 August 1978, *I. Trinajstić s.n.* (CNHM4058:BOT); Dubrovnik, Lapad, 9 October 1977, *I. Trinajstić s.n.* (CNHM4050:BOT); Srđ iznad Dubrovnika, 29 November 1978, *I. Trinajstić s.n.* (CNHM4051:BOT). **Bosnia and Herzegovina.** Weideplätze – V. Trebinje, n. Lastva, 7 August 1895, *Matulić s.n.* (SARA44076); In rupestribus montis Gliva prope Trebinje, July 1891, *K. Vandas s.n.* (SARA44075); Trebinje, 19 July 1892, *E. Brandis s.n.* (SARA44070); Am Wege von Meka gruda nach Djeć, 20 July 1925, *V. Hawelka s.n.* (SARA44054); In saxosis apricis prope Zavala, 18 October 1909, *K. Malý s.n.* (SARA44052). **Montenegro.** Katunska nahija, 2 August 1991, *V. Stevanović s.n.* (BEOU1578.91); Ad Viluša, ca. 1.100 m, July 1904, *J. Rohlena s.n.* (PI012665); vidikovac iznad jezera Slano, 31 July 2021, *S. Bogdanović s.n.* (CAT, ZAGR); Gruda, Majden, 13 July 1928, *D. Petrović s.n.* (BEOU37275); Orjen, Poštirovnik, s. Prčanj cca 890 m s.m., s. dol., 2 August 1980, *Č. Šilić s.n.* (SARA44071, 44072, 44073, 44074). ***Centaureadivergens*: Croatia.** Island of Hvar, St. Nikola, rocky grassland, 28 July 2021, *S. Bogdanović s.n.* (CAT, ZAGR); Zadvarje, along the road, 28 July 2021, *S. Bogdanović s.n.* (CAT, ZAGR); In apricis saxos. ins. Lesina, *Visiani s.n.* (P02815387); Dalmatia, in lapidos., pr. Macarsca, July 1880, *Th. illeg. s.n.* (P02815388); In collibus apricis Ins. Lessina, *D. Lagger s.n.* (P02815389); Lesina, *s.l., s.n.* (P02815389); Otok Šipan, ograde u garigu, 9 June 1979, *M. Hećimović s.n.* (ZA13512); Srednja Dalmacija, Grabovac, istočno od Šestanovca, uz cestu, 13 July 2014, *S. Bogdanović & U. Buzurović s.n.* (ZAGR38697, ZAGR38698); Grabovac, krečnjak, 13 July 2014, *U. Buzurović & S. Bogdanović s.n.* (BEO); Dalmacija, poluotok Pelješec, uvala Marčuleti, Rt Vrba, okomite stijene uz cestu, *M. Jeričević, N. Jeričević & S. Bogdanović s.n.* (ZAGR43796); Dalmatinska Zagora, Vrgorac, Prapatnice, Vegari, 20 July 20215, *M. Vukojević s.n.* (ZAGR40083); Neretva, okolica mjesta Rogotin, ruderalno, uz cestu, 20 July 2013, *S. Bogdanović & I. Boršić* (ZAGR44348); op keinestrand b. Makarska, 26 July 1965, *T. Baretta 4* (U1112735); Dalmatia. In agris sterilibus ad Macarscam, *s.d.*, *Pichler s.n.* (P02815392, P02815393, MW0794555, BEOU37201); Dalmatia, Lessina, *s.d.*, *M. Botteri s.n.* (BEOU37200); Mala Duba, Živogošće, kamenite padine uz Jadransku magistralu, 12 July 2003, *S. Maslo s.n.* (ZAGR); Brač, Vidova gora, October 1976, *I. Trinajstić s.n.* (CNHM4054:BOT); Otok Brač, kod Bola, 28 March 1969, *I. Trinajstić s.n.* (CNHM4053:BOT); Otok Brač, uz cestu Supetar – Nerežišće, May 1968, *I. Trinajstić s.n.* (CNHM4052:BOT). **Bosnia and Herzegovina.** Bosna, između Rilje i Kifinog sela, July 1969, *I. Trinajstić s.n.* (CNHM4055:BOT); Mostar, am Humberg, 100 m, July 1903, *E. Sagorski s.n.* (P04453688, P04453689); Mostar, in incultis, July 1907, *E. Sagorski s.n.* (P04132171); Mostar, in incultis ad m. Humberg, July 1905, *E. Sagorski s.n.* (SARA44056); Ad vias, July 1908, *E. Sagorski s.n.* (PI12696); Flora Hercegovine, Prenj, Porim, na izloženom grebenu, 29 July 1962, *I. Horvat s.n.* (ZAHO); Žitomislići, Mostar, kamenite padine i šikare brda Osojnica, July 2001, *S. Maslo s.n.* (ZAGR); Hercegovina, Prenj pl., Prevoj, kamenite padine uz put za Rujište, July 2011, *S. Maslo s.n.* (ZAGR); Stolac, Križevac, 260 m alt., 43.06159N, 17.9839E, kamenjari, krečnjak, 1 July 2015, *M. Niketić, G. Tomović, K. Jakovljević & S. Đurović s.n*. (BEOU43550); In locis siccis agris Livanjsko polje, prope Prolog, 22 June 1970, *H. Ritter s.n.* (SARA48730); In locis siccis agris Livanjsko polje, prope Kablić, 30 July 1970, *H. Ritter s.n.* (SARA48729); In valle Drežanka prope Drežnica, 4 August 1900, *K. Malý s.n.* (SARA 44058); In paucibus, Narontis, prope g. Grabovica, ca. 150 m, 5 October 1930, *K. Malý s.n.* (SARA44057); In paucibus, Narontis, prope g. Grabovica, 140 m, 7 September 1907, *K. Malý s.n.* (SARA44050); Konjic, in monte Vitaljica et Zlatar, 27 June 1955, *H. Ritter s.n.* (SARA44055); In valle Narontis prope Glavatičevo, solo dolomitico, 380 m, 17 July 1923, *K. Malý s.n.* (SARA44053); Inter Podgorjani-Lišani, 260 m, 18 August 1918, *K. Malý s.n.* (SARA44051); In saxosis apricis prope Bišina, ca. 1000 m, 7 August 1907, *K. Malý s.n.* (SARA44049); U dolini Rakitnice ispred Blaca, 10 August 1957, *H. Ritter s.n.* (SARA48725). **Montenegro.** Boka Kotorska, Luštica iznad Rosa, kamenjari sa *Salviaofficinalis*, krečnjak, 100 m, 3 August 1996, *D. Lakušić & B. Lakušić s.n.* (BEOU2150/96); Boka Kotorska, Luštica, prevoj izmedju Rosa i Kraćića, kamenjari, krečnjak, 350 m, 24 August 2002, *D. Lakušić & B. Lakušić s.n.* (BEOU15337); Kotor, put Prčanj-Rose, 29 June 2003, *P. Janaćković s.n*. (BEOU38399); Iznad Vrbanja, kamenjari sa *Globulariacordifolia*, 16 June 1990, *V. Stevanović s.n.* (BEOU1198.90); Klinci, Sv. Tripun, Luštica, kamenje pored šoderskog put, 24 June 1995, *V. Karaman 501* (BEOU37273).

### ﻿Key to *Centaurealovricii* and allied species

**Table:** 

1	Stem with 7–30 capitula; basal leaves fleshy, shiny, leaflets lanceolate to linear (0.8–10 mm wide); involucre 13–15 mm long, 8–12 mm wide; phyllaries up to 15 mm long, appendages dark-brown, fimbriae dense; outermost florets with lobes 7.5–11.5 mm long; pappus bristles up to 2.3 mm long	***C.lovricii***
–	Stems with up to 80 capitula; basal leaves rigid, dull, leaflets linear-filiform (0.5–2 mm wide); involucre 6–10 mm long, 4–6.5 mm wide; phyllaries up to 8 mm long, appendages straw-coloured tinged with pale brown, fimbriae sparse; outermost florets with lobes 2–6 mm long; pappus up to 1.1 mm long	**2**
2	Leaves glabrous; fimbriae on phyllaries up to 1(–1.5) mm long; disc floret tube 5–6 mm long and lobes 2–2.5 mm long; pappus bristles up to 0.8 mm long	***C.glaberrima***
–	Leaves hispid; fimbriae on phyllaries up to 2 mm long; disc floret tube 6.5–7.5 mm long and lobes 3–3.3 mm long; pappus bristles up to 1.1 mm long	***C.divergens***

## Supplementary Material

XML Treatment for Centaurea lovricii

XML Treatment for Centaurea glaberrima

XML Treatment for Centaurea divergens
